# Long-term survival and second malignant tumor prediction in pediatric, adolescent, and young adult cancer survivors using Random Survival Forests: a SEER analysis

**DOI:** 10.1038/s41598-023-29167-x

**Published:** 2023-02-02

**Authors:** Ivy Y. Zhang, Gregory R. Hart, Bo Qin, Jun Deng

**Affiliations:** 1 Department of Statistics and Data Science, Yale University, New Haven, CT USA; 2 Institute for Disease Modeling, Global Health Division, Bill & Melinda Gates Foundation, Seattle, WA USA; 3 Department of Quantitative Biomedical Science, Dartmouth College, Hanover, NH USA; 4 Department of Therapeutic Radiology, Yale University, New Haven, CT USA

**Keywords:** Cancer models, Statistics, Cancer epidemiology

## Abstract

Survival and second malignancy prediction models can aid clinical decision making. Most commonly, survival analysis studies are performed using traditional proportional hazards models, which require strong assumptions and can lead to biased estimates if violated. Therefore, this study aims to implement an alternative, machine learning (ML) model for survival analysis: Random Survival Forest (RSF). In this study, RSFs were built using the U.S. Surveillance Epidemiology and End Results to (1) predict 30-year survival in pediatric, adolescent, and young adult cancer survivors; and (2) predict risk and site of a second tumor within 30 years of the first tumor diagnosis in these age groups. The final RSF model for pediatric, adolescent, and young adult survival has an average Concordance index (C-index) of 92.9%, 94.2%, and 94.4% and average time-dependent area under the receiver operating characteristic curve (AUC) at 30-years since first diagnosis of 90.8%, 93.6%, 96.1% respectively. The final RSF model for pediatric, adolescent, and young adult second malignancy has an average C-index of 86.8%, 85.2%, and 88.6% and average time-dependent AUC at 30-years since first diagnosis of 76.5%, 88.1%, and 99.0% respectively. This study suggests the robustness and potential clinical value of ML models to alleviate physician burden by quickly identifying highest risk individuals.

## Introduction

In 2022 in the United States, about 10,500 pediatrics (under age 15), 5500 adolescents (age 15–19), and 80,000 young adults (age 20–39) are expected to be diagnosed with cancer^1–3^. Prior to 1970, cancer survival rates were bleak; for example, almost all pediatric cancer patients died as a result of their primary disease^4^. Fortunately, in the last decades, advances in early detection, treatments, and supportive care have expanded the population of cancer survivors^5^.

The most commonly reported measure of cancer survival is the 5-year survival rate^6^. However, as the number of childhood, adolescent, and young adult survivors grows, there is an increasing need and interest to investigate long-term outcomes. In addition to examining long-term survival of this population, clinicians are also interested in understanding the development of secondary malignant tumors as a late effect for their treatment decision making^7^. This is because the risk of second malignancies in cancer patients is elevated by primary cancer treatments such as chemotherapy or radiotherapy^8^.

Multivariate survival analyses with censored data are most commonly performed, particularly in medical research, using traditional proportional hazards models such as the Cox regression in combination with nomograms^9^. The Cox model’s advantages include not needing to define a distribution for the survival time and high interpretability in the form of a regression between survival time and explanatory variables^10^. Several recent studies with the U.S. Surveillance Epidemiology and End Results (SEER) data using this method developed cancer-type specific prediction models for pediatric, adolescents, and young adults for 3-, 5-, and 10-year overall survival^11–13^. Second malignancy risks were also investigated with this method in studies of 3- and 5-year risks after prostate cancer and 1-year risk after rectal cancer radiation therapy^14^.

However, one of the limitations of applying the Cox model is the assumption of proportional hazard (constant hazard ratio for any two individuals over time), which is often violated in high dimensional covariates^15^. One alternative to predicting time-to-events data is turning the problem into a classification one without censored data. For example, one recent study applied artificial neural networks to SEER data to predict, with 95% test accuracy, which thyroid cancer patients had survived more than 10 years since diagnosis versus passed away within the first 5 years^16^. Other SEER-based studies recently classified 6-month, 9-month, 1-, 2-, and 5-year lung cancer survival with various models such as decision trees and neural network^17^. Outside of SEER data, other recent studies that achieved high performance from this method include Random Forest (RF) prediction of breast cancer recurrence within 5 and 10 years, with 88% area under the receiver operating characteristic curve (AUC) on the test set^18^. While this method avoids the complications of dealing with censored data, useful insights can be lost when not evaluating information from censored instances^19^. Furthermore, ignoring censored outcomes can lead to severely biased estimates. Bias can also be introduced if censoring is not independent of covariates when using the hazard ratio^19–21^.

To address the challenges faced with modeling censored data and the restrictive proportional hazards assumption, recent innovations in machine learning (ML) techniques have been adapted. One such method is a survival tree ensemble model such as Random Survival Forest (RSF), a non-parametric approach that incorporates survival time and censoring information into the splitting criterion^22^. RSF is a variation of RF used on survival data. Just like RF, RSF is constructed from an ensemble of binary decision trees and each tree is built from a random bootstrap sample with the unsampled data used as an out-of-bag (OOB) data. A tree is built from its bootstrap sample through the process of random node splitting. At each split, *mtry* number of random variables are chosen as split candidates and one is chosen based on which maximizes the difference between the number of people who have reached an event vs those who have not at the time of the daughter nodes, similar to the impurity measure used in RF. The splitting process happens iteratively until all the uncensored events have happened and the tree is complete. The hazard function estimates, used for predicting survival at a point *t*, from each tree are calculated and then averaged over the whole forest to get the final estimate.

A few recent studies conducted with the SEER data employ this method for cancer-type specific survival and second malignancy prediction models: for example, one RSF model for predicting 4-year breast cancer survival status had a test AUC of 82.2% while one XGBoost model, another ensemble tree technique, had a test AUC of 91.1% for predicting 5-year survival status of osteosarcoma patients^23^.

While a few recent studies have been conducted to test ML methods to handle censored data with SEER, there have not been studies focusing on long term outcomes for these pediatric, adolescent, and young adult cancer survivors. This is a result of both insufficient longitudinal data and lower chances of long-term survival in the past prior to treatment advancements in the recent decades. Furthermore, these recent studies typically center around just one cancer type and do not include second tumor site prediction. Therefore, the goals of this study are twofold: (1) develop an ML model to predict 30-year survival in pediatric, adolescent, and young adult cancer survivors; and (2) develop an ML model to predict risk and site of a second tumor within 30 years of the first tumor diagnosis in these age groups.

## Methods

### The SEER dataset

The SEER dataset is one of the most comprehensive sources for U.S. cancer data, supported by the National Cancer Institute (NCI). SEER currently collects yearly data from various cancer registries, covering 47.9% of the U.S. population. The SEER November 2016 dataset used in this study includes patient demographics, primary tumor site, tumor morphology, and stage at diagnosis information between 1973 and 2014^24^. There are 134 variables present, including various recodes of the same information and year/site dependent breakouts of the same characteristic across several columns. The full data consists of 5,037,307 records, representing 4,370,772 unique patients. All data processing and modeling was completed using R version 4.0.2.

This study population includes patients whose first tumor record was before the age of 40 (pediatric, adolescent, or young adult at the time of first diagnosis). Only malignant tumor diagnosis records were included, as recommended by SEER for cancer analyses. Several steps were taken during data cleanup. First was reconciliation: columns representing the same characteristic, spread across different encodings or years were carefully combined into one using the most recent encoding schema available. For example, EOD—Tumor Size (representing years 1988–2003) and CS Tumor Size (representing years 2004+) were combined into one column based on the 2004+ CS sizing codes. Next, new variables necessary for the modeling were created, such as indicator columns for whether someone developed a 2nd, 3rd, or 4th+ tumor and whether the data for a given patient is censored.

Other data cleaning steps include re-encoding some factor variables as indicator columns, data transformations (e.g., log transformation of tumor size), and differentiated and consistent encoding of N/A versus missing values (e.g. whether the tumor stage for a patient’s 2nd tumor record was truly missing or whether this information is missing because a patient did not have a 2nd tumor). Finally, outlier records (e.g., outlier tumor sizes, 4th+ tumor record details) were excluded from the analyses. In addition, records whose sequence number had non-sequential diagnosis dates (e.g., record two had an earlier diagnosis date than record one) were also excluded as it was unclear whether this may have been a data entry issue or whether the record sequence does not reflect diagnosis date. The final dataset consists of 401,264 unique patient records. Detailed data-preprocessing steps can be found in Supplementary Table S1.

### Statistical methods and machine learning algorithms

Before modeling, imputation was used to handle the true missing values in the covariates. Multivariate Imputation by Chained Equations (MICE) with Categorical and Regression Trees (CART) from the MICE R package was compared with multiple imputation by RF from the missForest R package. MICE with CART imputes data, from leftmost to rightmost column, through sequential modeling, with CART modeling the distribution of each missing element conditioned on the other observed elements, until convergence of the models^25^. The package, missForest, imputes data iteratively, starting with the variable with the least missing observations to most, through fitting a RF on the observed values. This trained model is then used to predict the missing observation and the algorithm stops when the imputed data matrix converges^26^. In both cases, the assumption of missingness at random (MAR) was made. To assess accuracy, random values were removed from the set of complete cases in the variables of interest for imputation. Percentage of removed values mirrored the true missing proportions in the dataset to better mimic the missing data behavior of the data. Based on 100 iterations, missForest with mtry = 10 produce the highest average accuracy across the highest missing variables despite the larger variance in some cases, as seen in Supplementary Table S2 and Supplementary Fig. S1. One potential reason for the large difference in accuracy, despite the fundamental difference in the ordering of the imputation between the two algorithms, missForest implements Random Forest, which typically has better prediction accuracy than a decision tree given that it is an ensemble of trees. A detailed description of variables can be found in Supplementary Table S3.

The three main age groups of interest: pediatrics (n = 32,730), adolescents (n = 19,649), and young adults (n = 348,885), were separately modeled as cancer type prevalence and survival rates differ among the three groups. Table 1 shows the distributions of these three age groups in the final dataset. The imbalance in the proportion of censored records, particularly for the second tumor outcome, can pose a challenge to predictive modeling. This is because ML algorithms tend to consider minority class observations as outliers or noise, leading to lower predictive accuracy for the minority class^27^. One way to tackle this problem of imbalance is through balancing the dataset. In this study, synthetic minority oversampling technique (SMOTE) was used for the second tumor outcome RSF model. This technique oversamples from the minority classes by creating “synthetic” samples based on the k-nearest neighbors algorithm^28^. One-hot encoding from the mltools R package combined with 5-nearest neighbors SMOTE from the DMwR R package were adapted to balance the second tumor RSF model’s training data (85% of the data) to use for cross-validation. The test set was left untouched.

**Table 1:** Data distribution by age group.

Variable	Description	Pediatrics (n = 32,730)	Adolescents (n = 19,649)	Young adults (n = 348,885)
Site	Tumor site			
BREAST	Breast	0.03%	0.29%	11.83%
Colrect	Colon/rectum	0.21%	1.06%	3.03%
DIGOTHR	Other digestive	3.39%	1.22%	2.45%
FEMGEN	Female genital	1.36%	22.61%	35.13%
LYMLEUK	Lymphoma/leukemia	40.61%	29.17%	10.55%
MALEGEN	Male genital	1.04%	6.44%	5.36%
RESPIR	Respiratory	1.40%	1.35%	1.94%
URINARY	Urinary	6.24%	1.13%	2.04%
Other	All other sites	45.71%	36.73%	27.67%
	Total	100.00%	100.00%	100.00%
Censor	Censor (death)			
0	No	71.62%	76.30%	74.84%
1	Yes	28.38%	23.70%	25.16%
	Total	100.00%	100.00%	100.00%
Tumor.2	Censor (2nd tumor)			
0	No	96.81%	94.95%	90.46%
1	Yes	3.19%	5.05%	9.54%
	Total	100.00%	100.00%	100.00%
Death.30 yr	Died by year 30			
0	No	38.36%	36.37%	38.76%
1	Yes	61.64%	63.63%	61.24%
	Total	100.00%	100.00%	100.00%
Tumor2.30 yr	Second tumor by year 30			
0	No	97.16%	95.54%	91.23%
1	Yes	2.84%	4.46%	8.77%
	Total	100.00%	100.00%	100.00%

This study employs tree-based models, as opposed to deep learning and other more complex approaches, to explore the feasibility of long-term survival and second malignancy prediction in childhood, adolescent, and young adult cancer survivors for two main reasons: (1) physician skepticism of ML and (2) physician familiarity with decision trees. Firstly, the uptake of ML in medicine has been slow because they often fail to sufficiently show value for a clinician’s practice^29–31^. Therefore, one major consideration of this project is clinical value, especially since current researchers working on deployment of ML models in clinical environments typically do not take into account implementation and adoption barriers^32^. While in recent years, adoption of ML in the clinical workflow has increased, as seen by over 50 FDA cleared algorithms^33^, physicians often do not trust models they do not understand and many have very little familiarity with them^31,34–37^. Therefore, in order for a model to have some clinical value, it is necessary to consider approaches that are not overly-complex. Secondly, doctors and nurses are familiar with the concept of decision trees from their medical education^38,39^. While they do not think of it in technical terms as statisticians would of CART, for example, the concept of decision trees is something they use heuristically on a day-to-day basis when considering treatment pathways for patients. Therefore, when combined with the above point, an average of decision trees (in the form of RSF) would be more palatable and intuitive for healthcare providers who already have familiarity with decision trees compared to more complex methods such as neural networks.

To address the two questions of the study, RSF models were built on a training set, with time in months from diagnosis to death as the primary outcome for the survival model and time in months from diagnosis of first tumor to diagnosis of second tumor as the primary outcome for the second malignancy model. Supplementary Table S4 lists the final predictors included in the two RSF models. A fivefold cross validation (CV) grid search was performed to tune the mtry, split rule, and sample fraction of the RSF survival and second malignancy models respectively to identify an optimal model. The metric used in the CV assessment was the out-of-bag (OOB) concordance index (C-index). The C-index estimates the probability that the individual who reaches an event (e.g. death or second tumor) earlier has a higher predicted probability of reaching the event based on the RSF model^40,41^. This estimate is calculated by taking the proportion of all possible pairs of individuals, except those whose shorter survival time is censored or whose survival times are equal and censored, who are concordant, thereby assessing the overall performance of the model^22^.

Given that the SEER database is the most comprehensive cancer data in the US, representing over 30 registries and covering approximately 47.9% of the U.S. population, a separate cancer dataset spanning over 30 years in the US from registries not involved in SEER will be extremely difficult (if not impossible) to obtain. In order to overcome this data constraint and to ensure robustness of model results, 100 further iterations with random samples for training and testing were run and model performance metrics were averaged. This procedure of a repeated holdout test set (Monte Carlo Cross-Validation) produces a less overly optimistic (more conservative) and more stable estimate of the true performance of the model under any random test set^42^. In fact, this method may even overestimate the prediction error since only part of the data will be used for model training and calibration^43^.

In addition to the test C-index, time-dependent test area under the receiver operating curve (AUC) at 30-years was assessed using the R package, timeROC. The cumulative/dynamic time-dependent receiver operating curve (ROC) is calculated with inverse probability of censoring weighting (IPCW) estimation, which weights the cases and controls by the probability of being observed, estimated by the Kaplan–Meier (KM) estimator^44^. Unlike estimating sensitivity and specificity with empirical true positives and true negatives, this method does not assume that all subjects can be classified as cases or control and instead takes into account censored data.

The traditional Cox proportional hazards approaches were also implemented for modeling survival and second malignancy risk. This will allow us to understand how our proposed RSF method performs directly compared to a baseline approach. The assumption of proportional hazards using a score test of proportionality was conducted to confirm whether this method is appropriate. The null hypothesis tested is that the true population time-dependent coefficient of a covariate is 0, that is, the proportional hazards assumption is met. If this key assumption is not met, it can be concluded that the Cox regression may not be the best method as the results of the Cox regression could be biased, namely, the coefficient estimates and their interpretation are affected. The Cox models and score tests were run using the survival R package. Similar to the approach with the RSFs, 100 iterations were run, with varying train/test splits to get a more representative assessment of performance of the model given that randomness in train/test splits affect the final AUC. The final averaged C-index and time dependent AUC at 30-years was then calculated. One difference from the respective RSF counterparts of the Cox models is the variables modeled. Unlike the RSF, the Cox model coefficients are calculated using a maximum likelihood estimation approach, and convergence may not be achieved under certain scenarios such as in the presence of categorical variables with low incidence of one of the levels. Therefore, certain variables were excluded so that the Cox regression can be feasible, as detailed in Supplementary Table S5.

In addition to the RSF model for second malignancy prediction, RF models were trained to predict the site of second tumor as a next step in a two stage process. In the first stage: a binary classification RF was trained to predict whether a patient’s second tumor is “site specific” (BREAST, DIG_COLRECT, FEMGEN_MALEGN, LYMLYEUK, RESPIR, URINARY) or “non-site specific” (OTHER) as defined by SEER. Then, the second stage: a multiclass probability model further predicted the risk of different sites among the “site specific” second tumor patients. This two-stage approach enabled better model training in the face of an imbalanced multiclass classification problem, since the majority of patient sites in the SEER data were marked “OTHER”. Supplementary Table S6 lists the final predictors included in the RF models for second tumor site prediction. Like the RSF models for survival and second malignancy, a fivefold cross validation (CV) grid search was performed, this time to tune the mtry, number of trees, minimum node size, split rule, and sample fraction. CV accuracy in the training set was assessed for final model selection. A generalized multiclass AUC metric as defined by Hand et al.^45^ was reported based on an average of 100 further iterations. Details on the hyperparameter tuning across all models including the range of searches (limited due to available computational resources) and final chosen parameters can be seen in Table 2.

**Table 2:** Hyperparameters for RSF and RF.

Parameter	Description	Survival (RSF)			
		Range	Selected		
			Pediatrics	Adolescents	Young adult
mtry	Number of candidate variables	1–15	4	4	5
num.trees	Number of trees	50, 60, 70, 80, 90, 100, 350, 400	50	80	100
min.node.size	Minimum node size	1, 5, 10, 15–20	20	1	10
splitrule	Split rule	c, logrank	logrank	logrank	logrank
sample.fraction	Sample fraction	0.632, 1	0.632	0.632	0.632

Parameter	Description	Second malignancy risk (RSF)			
		Range	Selected		
			Pediatrics	Adolescents	Young adult
mtry	Number of candidate variables	2–8	6	6	7
num.trees	Number of trees	100, 200, 300	200	200	100
min.node.size	Minimum node size	1, 10	1	1	10
splitrule	Split rule	c, logrank	logrank	logrank	logrank
sample.fraction	Sample fraction	0.632, 1	0.632	0.632	0.632

Parameter	Description	Second malignancy site (RF-binary)			
		Range	Selected		
			All		
mtry	Number of candidate variables	2, 3, 4, 5, 8, 10, 15	4		
num.trees	Number of trees	200, 300, 350, 400, 450, 500, 600, 700, 800	350		
min.node.size	Minimum node size	10, 15, 20, 25, 26	26		
splitrule	Split rule	gini, extratrees, hellinger	gini		
sample.fraction	Sample fraction	0.632, 1	0.632		

Parameter	Description	Second malignancy site (RF-multiclass)			
		Range	Selected		
			All		
mtry	Number of candidate variables	5, 8, 10, 15	8		
num.trees	Number of trees	400, 450, 500	500		
min.node.size	Minimum node size	10, 15, 20, 30, 40	40		
splitrule	Split rule	gini	gini		
sample.fraction	Sample fraction	0.632, 1	0.632		

**Figure 1:**
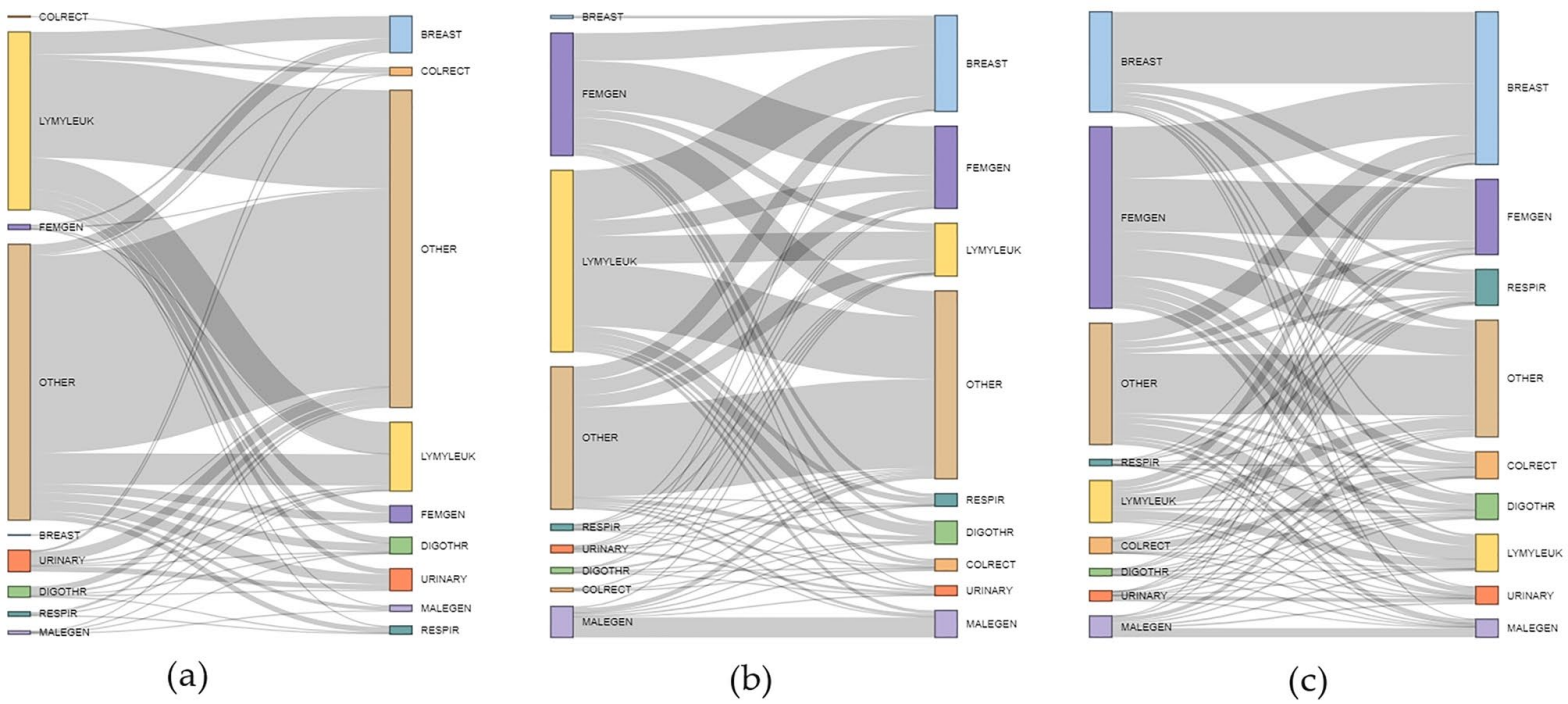
(**a**) Pediatric first (left) to second (right) tumor site within 30 years of first diagnosis (n = 930); (**b**) Adolescent first (left) to second (right) tumor site within 30 years of first diagnosis (n = 876); (**c**) Young adult first (left) to second (right) tumor site within 30 years of first diagnosis (n = 30,595).

Unlike the RSF models, the RF models for predicting the site of a second tumor were not broken out into pediatrics, adolescents, and young adults due to small sample from the pediatric and adolescent group for most sites. Furthermore, unlike for survival, SMOTE could not have been applied here because there are multiple minority classes; the SMOTE algorithm is only applicable for binary class imbalance. However, age group (pediatric, adolescent, young adult) was added as a variable to capture differences in distribution of tumor sites, as seen in Fig. 1. This Sankey diagram of patients who develop a second tumor within 30 years of first diagnosis is a network graph that illustrates the proportional distribution of first and second tumor sites. These sites are represented by each node on the left and right respectively. The flow illustrates the proportion of patients who start at one tumor site and then develop a second. This diagram illuminates the plethora paths a patient’s development can take, further increasing the need for a predictive model that can help a clinician better pinpoint second tumor location.

## Results

### Cox regression for modeling survival

The proportional hazards assumption was checked for each respective case of pediatric, adolescent, and young adult survival. Supplementary Table S7 shows a summary of the results of the score test for proportionality for the 100 iterations.

**Figure 2:**
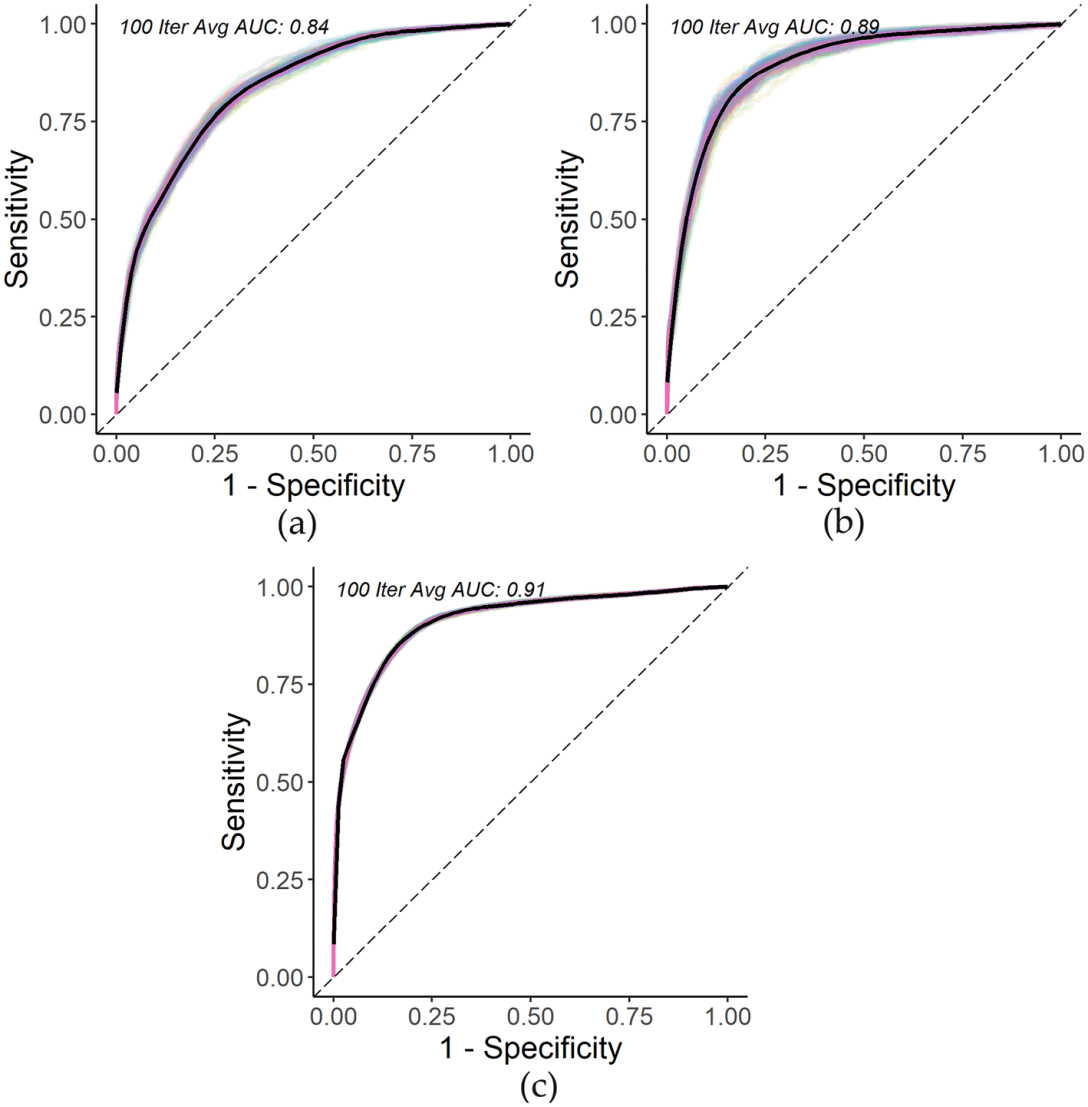
(**a**) Pediatric 30-year Cox survival time-dependent ROC curve over 100 iterations; (**b**) Adolescent 30-year Cox survival time-dependent ROC curve example over 100 iterations; (**c**) Young adult 30-year Cox survival time-dependent ROC curve over 100 iterations.

For almost all the covariates, we reject the null hypothesis that there is a constant hazard ratio over time given the small p-values. Therefore, the Cox regression is, in fact, not an appropriate model to use as we cannot reliably interpret the coefficient results per usual due to potential bias. In fact, despite the Cox regression typically being favored for its interpretability, this advantage no longer holds if the proportional hazards assumption is not met. However, for the purpose of comparing prediction results of our proposed RSF method to a baseline method, since our goal is not inference, we proceeded with calculating the Cox regression coefficients and assessing the predictive performance at 30-years since first diagnosis. The following are the averaged results across the 100 iterations: pediatric C-index of 80.0% (standard deviation of 0.6%) and time-dependent AUC at 30-years since first diagnosis of 83.7% (standard deviation of 0.6%), adolescent C-index of 85.3% (standard deviation of 0.7%) and time-dependent AUC at 30-years since first diagnosis of 89.4% (standard deviation of 0.9%), YA C-index of 85.3% (standard deviation of 0.2%) and time-dependent AUC at 30-years since first diagnosis of 91.3% (standard deviation of 0.2%). Figure 2 illustrates the average time-dependent test AUC at 30-years since first diagnosis of the three age groups across 100 iterations.

### Cox regression for modeling development of second tumor

Similarly, we evaluate the hazard ratio over time for the second tumor development model for pediatrics, adolescents, and young adults. Based on the results of the score test seen in Supplementary Table S8 we reject the null hypothesis that there is a constant hazard ratio over time for most covariates; the Cox regression’s typical coefficient interpretations may be biased and unreliable.

**Figure 3:**
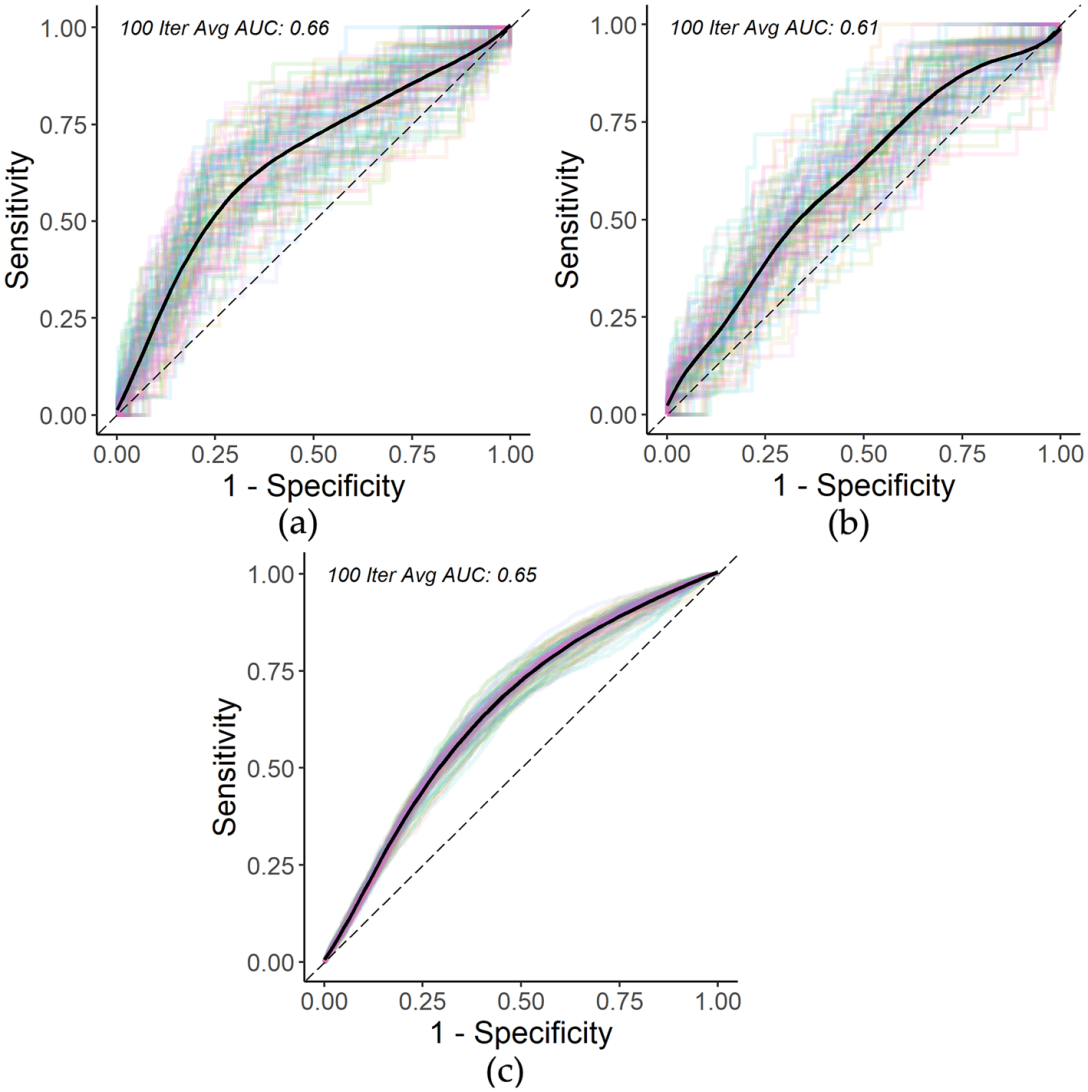
(**a**) Pediatric 30-year Cox second tumor time-dependent ROC curve over 100 iterations; (**b**) Adolescent 30-year Cox second tumor time-dependent ROC curve over 100 iterations; (**c**) Young adult 30-year Cox second tumor time-dependent ROC curve over 100 iterations.

However, for our prediction focused study, we build Cox models to likewise model the risk of second tumor development over time to compare with our proposed RSF model’s predictive performance. The averaged results over 100 iterations are as follows: pediatric C-index of 68.9% (standard deviation of 2.4%) and time-dependent AUC at 30-years since first diagnosis of 65.8% (standard deviation of 5.5%), adolescent C-index of 70.0% (standard deviation of 2.5%) and time-dependent AUC at 30-years since first diagnosis of 60.9% (standard deviation of 6.4%), YA C-index of 63.5% (standard deviation of 0.8%) and time-dependent AUC at 30-years since first diagnosis of 64.6% (standard deviation of 1.6%). Figure 3 presents the time-dependent test AUC at 30-years since first diagnosis of the three age groups. The high standard deviation across results, particularly among pediatrics and adolescents from different test/train splits suggests a high variance in the data as it relates to second tumor development, and therefore a higher difficulty of predicting second tumor risk. It further highlights the importance of the 100 iterations we ran for robustness in results when reporting average AUC and C-index for this context. While the standard deviation across results for young adults is not small, there is less variance as it relates to second tumor development as compared to younger age groups.

### Random Survival Forest for modeling survival

**Figure 4:**
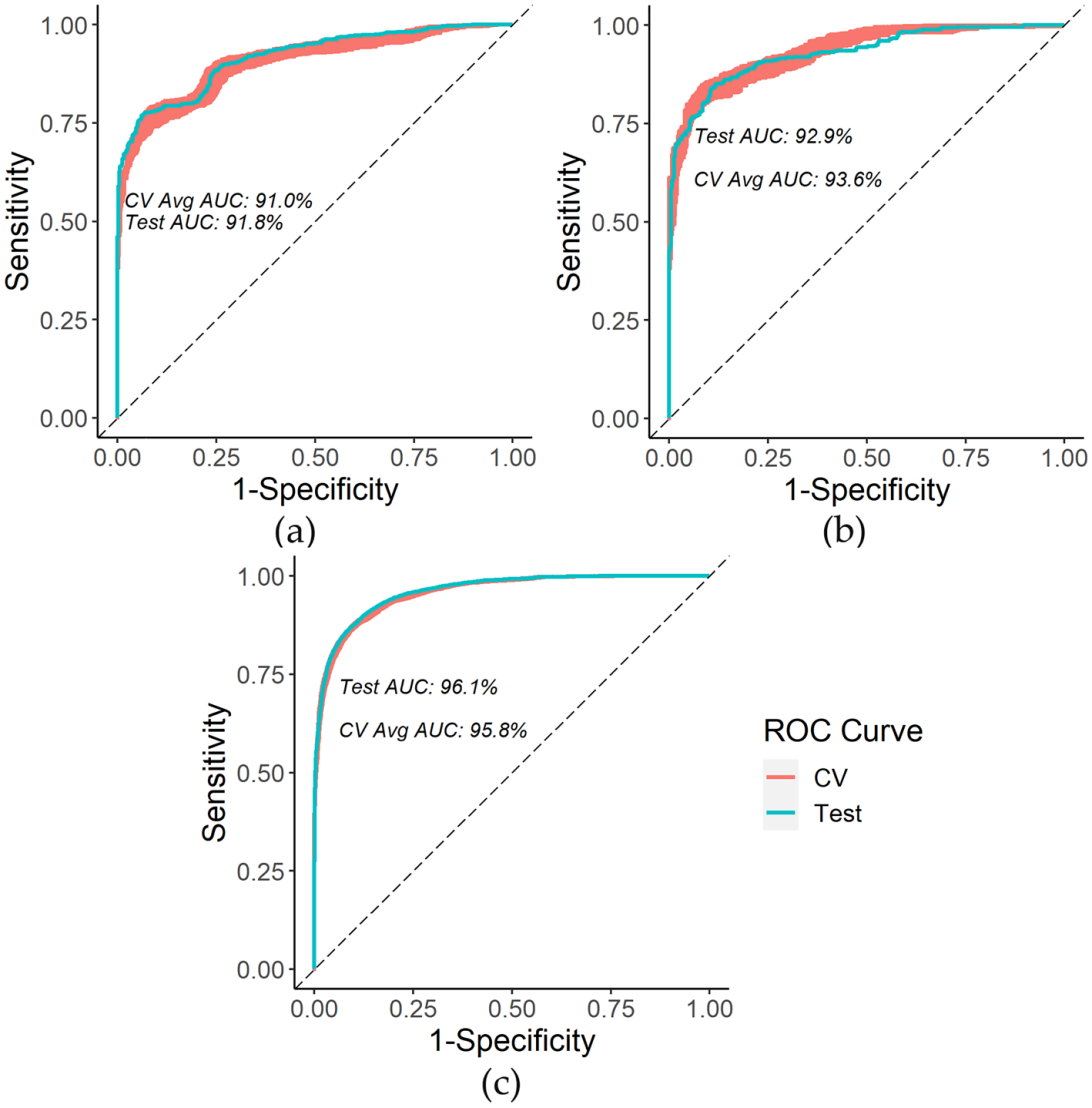
(**a**) Pediatric 30-year survival time-dependent ROC curve example; (**b**) Adolescent 30-year survival time-dependent ROC curve example; (**c**) Young adult 30-year survival time-dependent ROC curve example.

Firstly, based on the final selected RSF for modeling survival per the CV grid search, the three survival models have the following average test metrics based on 100 iterations: pediatric C-index of 92.9% (standard deviation of 0.4%) and time-dependent AUC at 30-years since first diagnosis of 90.8% (standard deviation of 1.0%); adolescent C-index of 94.2% (standard deviation of 0.2%) and time-dependent AUC at 30-years since first diagnosis of 93.6% (standard deviation of 0.8%); YA C-index of 94.4% (standard deviation of 0.09%) and time-dependent AUC at 30-years since first diagnosis of 96.1% (standard deviation of 0.2%). Compared to the corresponding Cox model, this represents an improvement of 12.9% in C-index and 7.1% in time-dependent AUC at 30-years since first diagnosis for pediatrics, 8.9% in C-index and 4.2% in time-dependent AUC at 30-years since first diagnosis for adolescents, and 9.1% in C-index and 4.8% in time-dependent AUC at 30-years since first diagnosis for young adults. Figure 4 presents the AUC range of fivefold CV and test AUC for the first iteration of the 30-year RSF survival for the three age groups.

### Random Survival Forest for modeling development of second tumor

**Figure 5:**
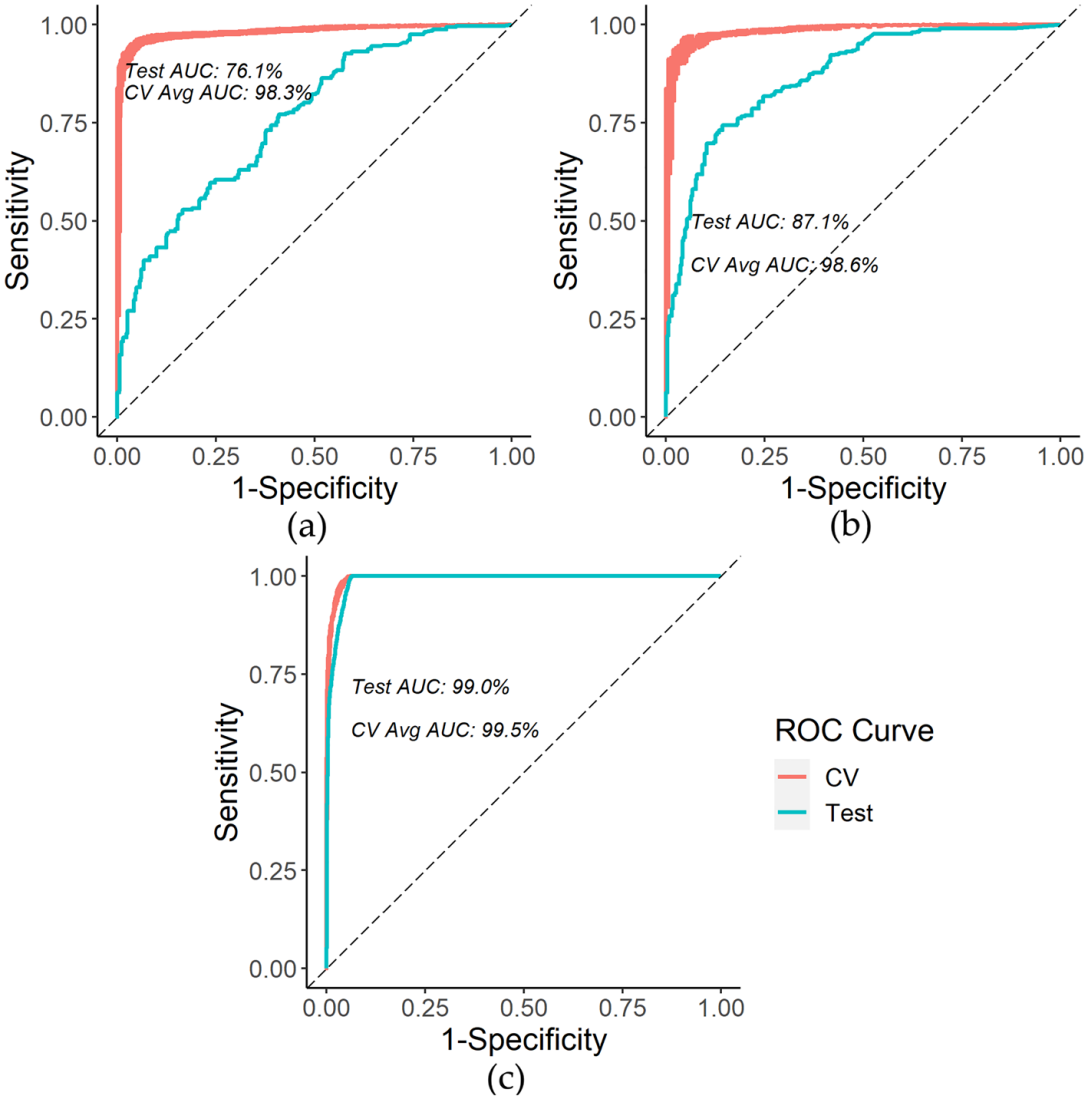
(**a**) Pediatric 30-year second tumor time-dependent ROC curve example; (**b**) Adolescent 30-year second tumor time-dependent ROC curve example; (**c**) Young adult 30-year second tumor time-dependent ROC curve example.

Secondly, the three final second malignant tumor RSF models have the following average test metrics based on 100 iterations: pediatric C-index of 86.8% (standard deviation of 0.4%) and time-dependent AUC at 30-years since first diagnosis of 76.5% (standard deviation of 2.3%); adolescent C-index of 85.2% (standard deviation of 0.7%) and time-dependent test AUC at 30-years since first diagnosis of 88.1% (standard deviation of 1.7%); YA C-index of 88.6% (standard deviation of 0.2%) and time-dependent AUC at 30-years since first diagnosis of 99.0% (standard deviation of 0.1%). This is a large improvement in performance compared to the Cox regression for all age groups: 17.9% in C-index and 10.7% in time-dependent AUC at 30-years since first diagnosis for pediatrics, 15.2% in C-index and 27.2% in time-dependent AUC at 30-years for adolescents, and 25.1% in C-index and 34.4% in time-dependent AUC at 30-years since first diagnosis for young adults. Figure 5 presents the AUC range of fivefold CV and test AUC for the first iteration of the 30-year RSF for 30-year second malignant tumor prediction across the three age groups.

### Random forest for second tumor site classification

**Figure 6:**
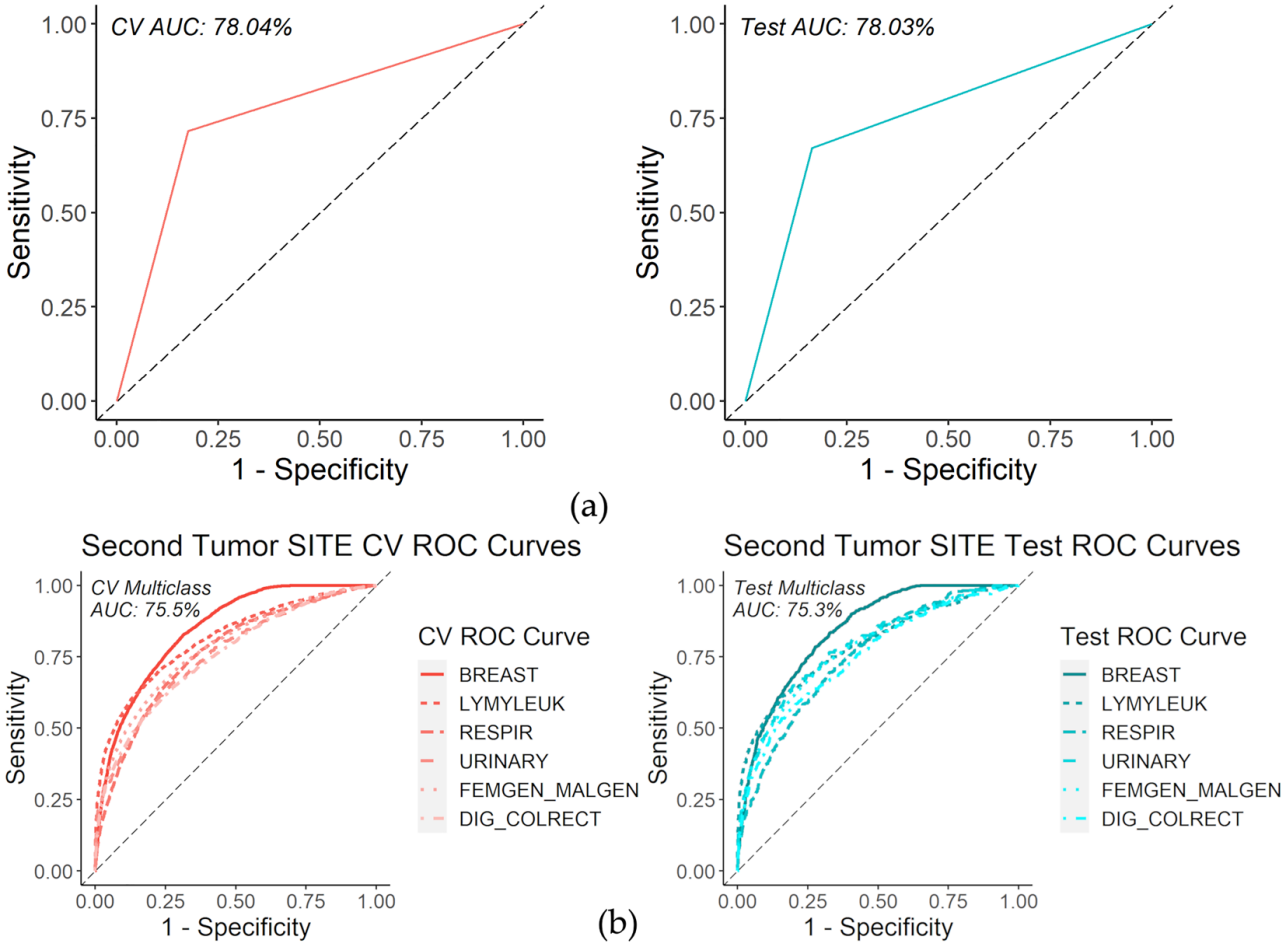
(**a**) Site specific vs non site specific ROC curve example. CV curve (left) and test curve (right); (**b**) Second tumor site ROC curve example. CV curve (left) and test curve (right).

Finally, the binary classification RF model for the first stage of the second tumor site prediction, site or non-site specific (“OTHER”), as defined by SEER tumor site classification, had an average test AUC of 78.2% (standard deviation of 0.7%), accuracy of 81.2% (standard deviation of 0.5%), average test positive predict value (PPV) of 70.6% (standard deviation of 2.8%), and average test negative predictive value (NPV) of 83.0% (standard deviation of 0.9%) from 100 iterations with random training and testing samples. The second stage of the second tumor site prediction (BREAST, DIG_COLRECT, FEMGEN_MALEGN, LYMLYEUK, RESPIR, or URINARY) involved a multiclass probability prediction. The final 6-class probability RF model had an average multiclass AUC of 75.9% (standard deviation of 0.5%) from 100 iterations. Figure 6 illustrates an ROC curve for the first and second stages of second tumor site prediction of the first iteration.

## Discussion

While time-to-event outcomes are typically evaluated at 5-years in most studies, with the availability of more healthcare data and improved medical treatments in recent decades that increased survival rates, long-term cancer survivor outcomes are meaningful to understand, particularly in younger age groups. The long-term outcome ML models developed in this study successfully predict survival and second tumor outcomes with high accuracy using easy to acquire, basic patient demographic and tumor information. All age groups’ RSF survival from first diagnosis had an average test C-index > 92% and all age groups’ RSF time-to-second malignancy from first diagnosis had an average test C-index > 86%, demonstrating the strength of ML extensions to traditional survival analysis, also seen in our comparison with respective Cox models’ performance. The comparison also illustrated that the interpretability of traditional Cox models, one of the big advantages compared to ML algorithms, is lost in our context because the key proportionality assumption does not hold for most covariates in this complex data. Furthermore, as compared to traditional prediction models such as Cox regressions for time-to-event modeling or logistic regression for classification, forest models do not require parametric or other strong assumptions. In addition, another strength of the models in this study is that they are not cancer specific and therefore more applicable to the general pediatric, adolescent, and young adult cancer survivor population.

There are several ways the models can be extremely useful to a clinician. Firstly, the survival model can help physicians identify high risk individuals among their many patients by quickly navigating through overwhelming quantities of EHR data. Furthermore, upon identifying patients with high risk of second malignancy, physicians can use that information in their treatment decision making process given that certain treatment types elevate chances of second malignancy. Finally, the RSF models built in this study can be used for subtype discovery—helping physicians learn about the subtypes among their cancer patients. For example, if two patients are “close” in terms of RF proximity, their outcome and features are similar. Methods such as community detection can then be applied to cluster on the proximity space and uncertainty can be characterized based on proximity to the training data.

However, the present study has some limitations. First, some vital longitudinal patient data linked to survival and second tumor development such as socioeconomic status, treatment history, or comorbidities were not available for modeling. Therefore, further studies can benefit from gathering additional data on clinically relevant variables. Next, while tree-based methods such as missForest, RF, and RSF can achieve high accuracy, the algorithms build trees that suffer from high variance, although the ensemble can help reduce some variance^46^. Finally, one of the assumptions of the KM based estimation used in the ROC plots is independence between covariates and censoring. Therefore, the variable year of first diagnosis, may have introduced bias to the AUROC estimate since those who were diagnosed after 1984 are censored by definition. Further work can explore adapting the existing time-dependent ROC R package to use estimators without such an assumption such as censoring-robust estimation of AUC^47^ or the doubly-robust censoring unbiased transformation^48^ instead of IPCW.

In future studies, additional patient level features from disparate sources can benefit continued work in survival and second malignant tumor prediction by tackling some of the aforementioned limitations. In addition, collaborations with practicing physicians would be an important component in improving prediction models to further increase clinical value of ML applied in practice. Furthermore, as the RSF extension of the RF exhibited, there is high potential for testing more survival analysis extensions of current ML algorithms.

As final remarks, the present study illustrated the ability of RSF and a multistage RF to predict 30-year survival and second malignant tumor risk and site in pediatric, adolescent, and young adult cancer survivors. These models demonstrated strong performance based on AUC. In the future, survival and second tumor models such as the ones developed in this study could help physicians navigate overwhelming quantities of patient data by quickly identifying highest risk individuals and ultimately improving young cancer survivor outcomes.

## Supplementary Information

Supplementary Information.

## Data Availability

The SEER data set used in this publication is not owned by the authors, but by the National Cancer Institute. While we are not allowed to share the data, any interested party can apply for access from the National Cancer Institute (https://seer.cancer.gov/data/access.html). The corresponding author is also willing to share his experience and advice in applying for access if needed.
