# Supplementary material for: Long-term survival and second malignant tumor prediction in pediatric, adolescent, and young adult cancer survivors using Random Survival Forests: a SEER analysis
Source: Sci Rep. 2023 Feb 2;13:1911. doi: 10.1038/s41598-023-29167-x (PMC9894907; doi:10.1038/s41598-023-29167-x)
Supplement: Supplementary file 1 — Supplementary Information. [file 41598_2023_29167_MOESM1_ESM.docx]

# Long-term survival and second malignant tumor prediction in pediatric, adolescent, and young adult cancer survivors using Random Survival Forests: a SEER analysis

**Ivy Y. Zhang^1^, Gregory R. Hart^2^, Bo Qin^3^, Jun Deng^4*^**

1 Department of Statistics and Data Science, Yale University, New Haven, CT, United States; ivy.zhang@aya.yale.edu

2 Institute for Disease Modeling, Global Health Division, Bill & Melinda Gates Foundation, Seattle, WA, USA; gregory.hart@gatesfoundation.org

3 Department of Quantitative Biomedical Science, Dartmouth College, Hanover, New Hampshire, United States; bo.qin.gr@dartmouth.edu

4 Department of Therapeutic Radiology, Yale University, New Haven, CT, United States; jun.deng@yale.edu

* Corresponding author: [jun.deng@yale.edu](mailto:jun.deng@yale.edu) (JD)

# Supplementary Tables and Figures

**Table S1:** Data pre-processing steps

|  |  |  |  |  |  |  |  |
| --- | --- | --- | --- | --- | --- | --- | --- |
|  |  |  |  |  |  |  |  |
|  | Description |  |  |  |  |  |  |
| 1. | Subset data to patients who were first diagnosed before age 40 |  |  |  |  |  |  |
| 2. | Combine SEER data across all sites into one database |  |  |  |  |  |  |
| 3. | Merge together some categories to reduce the levels in the following categorical variables |  |  |  |  |  |  |
|  | 1. CSTUMSIZ and EOD10_SZ |  |  |  |  |  |  |
|  | 2. DAJCCSTG and AJCC_STG |  |  |  |  |  |  |
|  | 3. SURGPRIF, SURGSCOF, SURGSITF, SURGSCOP, and SURGSITE |  |  |  |  |  |  |
|  | 4. CODPUBKM |  |  |  |  |  |  |
|  | 5. MAR_STAT |  |  |  |  |  |  |
| 4. | Merge togther the below variables into one |  |  |  |  |  |  |
|  | 1. CSTUMSIZ and EOD10_SZ as tumor_size |  |  |  |  |  |  |
|  | 2. CSEXTEN and EOD10_EX as tumor_ext |  |  |  |  |  |  |
|  | 2. CSLYMPHN and EOD10_ND as tumor_ln |  |  |  |  |  |  |
|  | 3. RAC_RECA and ORIGRECB as race_hisp |  |  |  |  |  |  |
|  | 4. DAJCCSTG and AJCC_STG as stage |  |  |  |  |  |  |
|  | 5. SURGPRIF, SURGSCOF, SURGSITF, SURGSCOP, and SURGSITE as surgery |  |  |  |  |  |  |
| 5. | Create 'censor' and age 'group' columns needed for modeling |  |  |  |  |  |  |
| 6. | Replace '9' or '999' (missing data) encodings with 'NA' for tumor related variables we will impute on |  |  |  |  |  |  |
| 7. | Log tumor size |  |  |  |  |  |  |
| 8. | Exclude patients who did not have any diagnosed malignant tumors |  |  |  |  |  |  |
| 9. | Reshape data to wide format |  |  |  |  |  |  |
| 10. | Remove outlier information (data points for 4+ tumors) |  |  |  |  |  |  |
| 11. | Remove inconsistent data such as: |  |  |  |  |  |  |
|  | 1. Age at first tumor diagnosis > age at second tumor diagnosis > age at third tumor diagnosis |  |  |  |  |  |  |
|  | 2. Year of first tumor diagnosis > year of second tumor diagnosis > year of third tumor diagnosis |  |  |  |  |  |  |

**Table S2:** Imputation Accuracy of Highest Missing Variables (>5% Missing)

| Variable  (>5% Missing) |  |  | % Accuracy (Categorical) | |  | MSE (Continuous) | |
| --- | --- | --- | --- | --- | --- | --- | --- |
|  | Description | % Missing | MICE (CART) | missForest |  | MICE (CART) | missForest |
| Categorical |  |  |  |  |  |  |  |
| GRADE | Tumor grade | 73% | 45% | 94% |  | - | - |
| SUMM2K | Tumor stage | 71% | 62% | 84% |  | - | - |
| surgery | Surgery (Yes / No) | 65% | 86% | 93% |  | - | - |
| Continuous |  |  |  |  |  |  |  |
| log_tumor_size | ln(tumor size) | 64% | - | - |  | 1.17 | 0.03 |
| pos_nodes | # Positive nodes | 34% | - | - |  | 12.38 | 4.65 |

**Table S3:** Final variables

|  | | |
| --- | --- | --- |
| Variables | | Description |
| Demographic | |  |
| 1. | mar_stat | Marital status throughout SEER documentation: 1 if single, 2 if married and never divorced or separated, 3 if ever divorced or separated |
| 2. | race_hisp | Patient's race and hispanic origin |
| 3. | SEX | Patient's sex at initial diagnosis |
| 4. | YR_BRTH | Patient’s year of birth |
| Overall Tumor | |  |
| 5. | MALIGCOUNT | Count of a patient’s total reported in situ/malignant cancers, based on maximum sequence number of any in situ/malignant first tumors in SEER through the last released year of diagnosis |
| 6. | BENBORDCOUNT | Count of a patient’s total reported benign/borderline cancers, based on maximum sequence number of any benign/borderline first tumors in SEER through the last released year of diagnosis |
| First Tumor | | |
| 7. | AGE_DX | Patient's age at diagnosis of first tumor |
| 8. | SUMM2K | Cancer stage of first tumor based on SEER Extent of Disease (EOD) following a SEER algorithm |
| 9. | MDXRECMP | The month the first tumor was first diagnosed by a recognized medical practitioner, whether clinically or microscopically confirmed |
| 10. | YEAR_DX | The year the first tumor was first diagnosed by a recognized medical practitioner, whether clinically or microscopically confirmed |
| 11. | GRADE | Grade of first tumor |
| 12. | LATERAL | The side of a paired organ or side of the body on which the reportable first tumor originated |
| 13. | SITE | Site group of first tumor |
| 14. | log_tumor_size | Natural logarithm of the largest dimension of the primary first tumor in millimeters |
| 15. | surgery | 1 if surgery was performed on the first tumor (surgery to remove/destroy tissue on primary site or remove distant lymph nodes/tissues/organs beyond primary site) |
| 16. | BEHTREND | Behavior of the first tumor: benign, borderline malignant, in situ, malignant |
| 17. | INSREC_PUB | Medical insurance status at time of first tumor |
| 18. | pos_nodes | Number of regional lymph nodes examined by the pathologist that were found to contain metastases at time of first tumor diagnosis |
| Second Tumor | |  |
| 19. | tumor.2 | 1 if patient has two or more tumors recorded, 0 otherwise |
| 20. | months_tumor.2 | Months between diagnosis of first and second tumor |
| 21. | AGE_DX.2 | Patient's age at diagnosis of second tumor |
| 22. | SUMM2K.2 | Cancer stage of second tumor based on SEER Extent of Disease (EOD) following a SEER algorithm |
| 23. | MDXRECMP.2 | The month the second tumor was second diagnosed by a recognized medical practitioner, whether clinically or microscopically confirmed |
| 24. | YEAR_DX.2 | The year the second tumor was second diagnosed by a recognized medical practitioner, whether clinically or microscopically confirmed |
| 25. | GRADE.2 | Grade of second tumor |
| 26. | LATERAL.2 | The side of a paired organ or side of the body on which the reportable second tumor originated |
| 27. | SITE.2 | Site group of second tumor |
| 28. | log_tumor_size.2 | Natural logarithm of the largest dimension of the primary second tumor in millimeters |
| 29. | surgery.2 | 1 if surgery was performed on the second tumor (surgery to remove/destroy tissue on primary site or remove distant lymph nodes/tissues/organs beyond primary site) |
| 30. | BEHTREND.2 | Behavior of the second tumor: Benign, Borderline malignancy, In situ, malignant |
| 31. | INSREC_PUB.2 | Medical insurance status at time of second tumor |
| 32. | pos_nodes.2 | Number of regional lymph nodes examined by the pathologist that were found to contain metastases at time of second tumor diagnosis |
| Third Tumor | |  |
| 33. | tumor.3 | 1 if patient has two or more tumors recorded, 0 otherwise |
| 34. | months_tumor.3 | Months between diagnosis of second and third tumor |
| 35. | AGE_DX.3 | Patient's age at diagnosis of third tumor |
| 36. | SUMM2K.3 | Cancer stage of third tumor based on SEER Extent of Disease (EOD) following a SEER algorithm |
| 37. | MDXRECMP.3 | The month the third tumor was third diagnosed by a recognized medical practitioner, whether clinically or microscopically confirmed |
| 38. | YEAR_DX.3 | The year the third tumor was third diagnosed by a recognized medical practitioner, whether clinically or microscopically confirmed |
| 39. | GRADE.3 | Grade of third tumor |
| 40. | LATERAL.3 | The side of a paired organ or side of the body on which the reportable third tumor originated |
| 41. | SITE.3 | Site group of third tumor |
| 42. | log_tumor_size.3 | Natural logarithm of the largest dimension of the primary third tumor in millimeters |
| 43. | surgery.3 | 1 if surgery was performed on the third tumor (surgery to remove/destroy tissue on primary site or remove distant lymph nodes/tissues/organs beyond primary site) |
| 44. | BEHTREND.3 | Behavior of the third tumor: Benign, Borderline malignancy, In situ, malignant |
| 45. | INSREC_PUB.3 | Medical insurance status at time of third tumor |
| 46. | pos_nodes.3 | Number of regional lymph nodes examined by the pathologist that were found to contain metastases at time of third tumor diagnosis |
| 47. | tumorfourplus | 1 if patient has four or more tumors recorded, 0 otherwise |

**Table S4:** Predictors for random survival forest models

|  |  |  |  |
| --- | --- | --- | --- |
| RSF - Time to Death | | RSF - Time to Second Tumor | |
| Demographic | | Demographic | |
| 1. | mar_stat | 1. | mar_stat |
| 2. | race_hisp | 2. | race_hisp |
| 3. | SEX | 3. | SEX |
| 4. | YR_BRTH | 4. | AGE_DX |
| Overall Tumor | | 5. | YR_BRTH |
| 5. | MALIGCOUNT | First Tumor | |
| 6. | BENBORDCOUNT | 6. | SUMM2K |
| First Tumor | | 7. | MDXRECMP |
| 7. | AGE_DX | 8. | YEAR_DX |
| 8. | SUMM2K | 9. | GRADE |
| 9. | MDXRECMP | 10. | LATERAL |
| 10. | YEAR_DX | 11. | SITE |
| 11. | GRADE | 12. | log_tumor_size |
| 12. | LATERAL | 13. | surgery |
| 13. | SITE | 14. | BEHTREND |
| 14. | log_tumor_size | 15. | INSREC_PUB |
| 15. | surgery | 16. | pos_nodes |
| 16. | BEHTREND |  |  |
| 17. | INSREC_PUB |  |  |
| 18. | pos_nodes |  |  |
| Second Tumor | |  |  |
| 19. | tumor.2 |  |  |
| 20. | months_tumor.2 |  |  |
| 21. | AGE_DX.2 |  |  |
| 22. | SUMM2K.2 |  |  |
| 23. | MDXRECMP.2 |  |  |
| 24. | YEAR_DX.2 |  |  |
| 25. | GRADE.2 |  |  |
| 26. | LATERAL.2 |  |  |
| 27. | SITE.2 |  |  |
| 28. | log_tumor_size.2 |  |  |
| 29. | surgery.2 |  |  |
| 30. | BEHTREND.2 |  |  |
| 31. | INSREC_PUB.2 |  |  |
| 32. | pos_nodes.2 |  |  |
| Third Tumor | |  |  |
| 33. | tumor.3 |  |  |
| 34. | months_tumor.3 |  |  |
| 35. | AGE_DX.3 |  |  |
| 36. | SUMM2K.3 |  |  |
| 37. | MDXRECMP.3 |  |  |
| 38. | YEAR_DX.3 |  |  |
| 39. | GRADE.3 |  |  |
| 40. | LATERAL.3 |  |  |
| 41. | SITE.3 |  |  |
| 42. | log_tumor_size.3 |  |  |
| 43. | surgery.3 |  |  |
| 44. | BEHTREND.3 |  |  |
| 45. | INSREC_PUB.3 |  |  |
| 46. | pos_nodes.3 |  |  |
| 47. | tumorfourplus |  |  |

**Table S5:** Variables/Factor Levels Excluded for Cox Regression Due to Low Incidence in Certain Levels

|  |  |  |  |  |  |  |
| --- | --- | --- | --- | --- | --- | --- |
|  | RSF - Time to Death | |  | RSF - Time to Second Tumor | | |
| Age Groups Excluded For | Variable | |  | Age Groups Excluded For |  | Variable |
|  |  |  |  |  |  |  |
| Pediatrics | First Tumor | |  | Pediatrics |  |  |
|  | 1. | BEHTREND |  |  | 1. | race_hisp level '2 1' |
| Pediatrics | Second Tumor | |  |  | 2. | LATERAL level '5' |
| Adolescents | 2. | months_tumor.2 |  |  | 3. | GRADE level '8' |
| Young Adult | 3. | AGE_DX.2 |  |  | 4. | INSREC_PUB level '1' |
|  | 4. | SUMM2K.2 |  |  | 5. | surgery levels 2-5 |
|  | 5. | MDXRECMP.2 |  |  | 6. | BEHTREND |
|  | 6. | YEAR_DX.2 |  | Adolescents | 1. | race_hisp level '2 1', '3 1', '99' |
|  | 7. | GRADE.2 |  |  | 2. | LATERAL level '5' |
|  | 8. | LATERAL.2 |  |  | 3. | GRADE level '7' and '8' |
|  | 9. | SITE.2 |  |  | 4. | INSREC_PUB level '1', '4', '5' |
|  | 10. | log_tumor_size.2 |  |  | 5. | surgery levels 2-5 |
|  | 11. | surgery.2 |  |  | 6. | BEHTREND |
|  | 12. | BEHTREND.2 |  |  |  |  |
|  | 13. | INSREC_PUB.2 |  |  |  |  |
|  | 14. | pos_nodes.2 |  |  |  |  |
|  | Third Tumor | |  |  |  |  |
|  | 15. | months_tumor.3 |  |  |  |  |
|  | 16. | AGE_DX.3 |  |  |  |  |
|  | 17. | SUMM2K.3 |  |  |  |  |
|  | 18. | MDXRECMP.3 |  |  |  |  |
|  | 19. | YEAR_DX.3 |  |  |  |  |
|  | 20. | GRADE.3 |  |  |  |  |
|  | 21. | LATERAL.3 |  |  |  |  |
|  | 22. | SITE.3 |  |  |  |  |
|  | 23. | log_tumor_size.3 |  |  |  |  |
|  | 24. | surgery.3 |  |  |  |  |
|  | 25. | BEHTREND.3 |  |  |  |  |
|  | 26. | INSREC_PUB.3 |  |  |  |  |
|  | 27. | pos_nodes.3 |  |  |  |  |

**Table S6:** Predictors for random forest model

|  |  |
| --- | --- |
| Demographic | |
| 1. | mar_stat |
| 2. | race_hisp |
| 3. | SEX |
| 4. | AGE_DX |
| 5. | YR_BRTH |
| First Tumor | |
| 6. | SUMM2K |
| 7. | MDXRECMP |
| 8. | YEAR_DX |
| 9. | GRADE |
| 10. | LATERAL |
| 11. | SITE |
| 12. | log_tumor_size |
| 13. | surgery |
| 14. | BEHTREND |
| 15. | INSREC_PUB |
| 16. | pos_nodes |

**Table S7:** Score Test Results – Time to Death

|  | | | | | | | |
| --- | --- | --- | --- | --- | --- | --- | --- |
| Cox - Survival | | | Pediatrics | | Adolescents | Young Adult |  |
| Demographic | |  | |  | |  |  |
| 1. | mar_stat | 0.000 (SD: 0.000) | | 0.000 (SD: 0.000) | | 0.000 (SD: 0.000) |  |
| 2. | race_hisp | 0.002 (SD: 0.005) | | 0.000 (SD: 0.000) | | 0.000 (SD: 0.000) |  |
| 3. | SEX | 0.026 (SD: 0.029) | | 0.013 (SD: 0.016) | | 0.000 (SD: 0.000) |  |
| 4. | YR_BRTH | 0.000 (SD: 0.000) | | 0.572 (SD: 0.215) | | 0.000 (SD: 0.000) |  |
| Overall Tumor | |  | |  | |  |  |
| 5. | MALIGCOUNT | 0.000 (SD: 0.000) | | 0.000 (SD: 0.000) | | 0.000 (SD: 0.000) |  |
| 6. | BENBORDCOUNT | 0.000 (SD: 0.000) | | 0.079 (SD: 0.108) | | 0.000 (SD: 0.000) |  |
| First Tumor | |  | |  | |  |  |
| 7. | AGE_DX | 0.000 (SD: 0.000) | | 0.028 (SD: 0.027) | | 0.000 (SD: 0.000) |  |
| 8. | SUMM2K | 0.000 (SD: 0.000) | | 0.000 (SD: 0.000) | | 0.000 (SD: 0.000) |  |
| 9. | MDXRECMP | 0.450 (SD: 0.196) | | 0.717 (SD: 0.193) | | 0.076 (SD: 0.077) |  |
| 10. | YEAR_DX | 0.000 (SD: 0.000) | | 0.773 (SD: 0.154) | | 0.739 (SD: 0.177) |  |
| 11. | GRADE | 0.000 (SD: 0.000) | | 0.000 (SD: 0.000) | | 0.000 (SD: 0.000) |  |
| 12. | LATERAL | 0.038 (SD: 0.049) | | 0.149 (SD: 0.117) | | 0.000 (SD: 0.000) |  |
| 13. | SITE | 0.000 (SD: 0.000) | | 0.000 (SD: 0.000) | | 0.000 (SD: 0.000) |  |
| 14. | log_tumor_size | 0.001 (SD: 0.003) | | 0.000 (SD: 0.000) | | 0.000 (SD: 0.000) |  |
| 15. | surgery | 0.000 (SD: 0.000) | | 0.000 (SD: 0.000) | | 0.000 (SD: 0.000) |  |
| 16. | BEHTREND | - | | 0.000 (SD: 0.000) | | 0.000 (SD: 0.000) |  |
| 17. | INSREC_PUB | 0.000 (SD: 0.000) | | 0.000 (SD: 0.001) | | 0.000 (SD: 0.000) |  |
| 18. | pos_nodes | 0.000 (SD: 0.000) | | 0.002 (SD: 0.002) | | 0.018 (SD: 0.023) |  |
| Second Tumor | |  | |  | |  |  |
| 19. | tumor.2 | 0.000 (SD: 0.000) | | 0.000 (SD: 0.000) | | 0.000 (SD: 0.000) |  |
| Third Tumor | |  | |  | |  |  |
| 20. | tumor.3 | 0.000 (SD: 0.000) | | 0.000 (SD: 0.000) | | 0.000 (SD: 0.000) |  |
| 21. | tumorfourplus | 0.000 (SD: 0.000) | | 0.031 (SD: 0.043) | | 0.000 (SD: 0.000) |  |

**Table S8:** Score Test Results – Time to Second Tumor

|  | | | |  |  |
| --- | --- | --- | --- | --- | --- |
| Cox - Second Malignancy Risk | | Pediatrics | Adolescents | | Young Adult |
| Demographic | |  |  | |  |
| 1. | mar_stat | 0.000 (SD: 0.000) | 0.000 (SD: 0.000) | | 0.000 (SD: 0.000) |
| 2. | race_hisp | 0.000 (SD: 0.000) | 0.000 (SD: 0.000) | | 0.050 (SD: 0.185) |
| 3. | SEX | 0.009 (SD: 0.082) | 0.069 (SD: 0.194) | | 0.006 (SD: 0.062) |
| 4. | AGE_DX | 0.018 (SD: 0.115) | 0.173 (SD: 0.292) | | 0.000 (SD: 0.000) |
| 5. | YR_BRTH | 0.000 (SD: 0.000) | 0.000 (SD: 0.000) | | 0.003 (SD: 0.026) |
| First Tumor | |  |  | |  |
| 6. | SUMM2K | 0.000 (SD: 0.000) | 0.000 (SD: 0.000) | | 0.000 (SD: 0.000) |
| 7. | MDXRECMP | 0.257 (SD: 0.339) | 0.003 (SD: 0.027) | | 0.004 (SD: 0.036) |
| 8. | YEAR_DX | 0.000 (SD: 0.000) | 0.000 (SD: 0.000) | | 0.000 (SD: 0.000) |
| 9. | GRADE | 0.000 (SD: 0.000) | 0.000 (SD: 0.000) | | 0.000 (SD: 0.000) |
| 10. | LATERAL | 0.000 (SD: 0.000) | 0.000 (SD: 0.000) | | 0.000 (SD: 0.000) |
| 11. | SITE | 0.000 (SD: 0.000) | 0.000 (SD: 0.000) | | 0.000 (SD: 0.000) |
| 12. | log_tumor_size | 0.000 (SD: 0.000) | 0.008 (SD: 0.070) | | 0.000 (SD: 0.000) |
| 13. | surgery | 0.001 (SD: 0.001) | 0.000 (SD: 0.000) | | 0.000 (SD: 0.000) |
| 14. | BEHTREND | 0.000 (SD: 0.000) | 0.000 (SD: 0.000) | | 0.000 (SD: 0.000) |
| 15. | INSREC_PUB | 0.000 (SD: 0.000) | 0.000 (SD: 0.000) | | 0.000 (SD: 0.000) |
| 16. | pos_nodes | 0.002 (SD: 0.014) | 0.001 (SD: 0.006) | | 0.000 (SD: 0.004) |


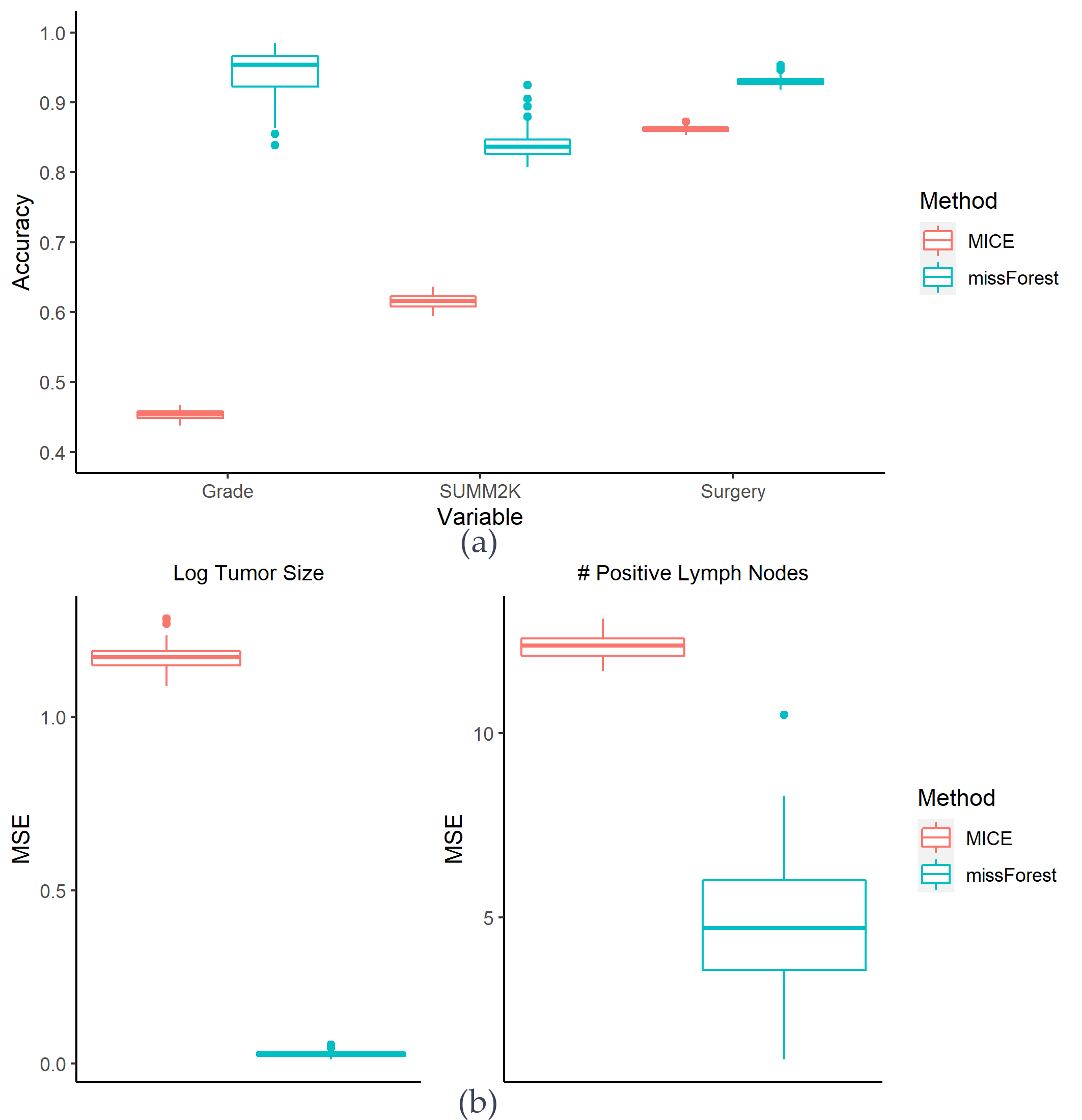
**Figure S1:** (**a**) Distribution of imputation accuracy of highest missing categorical variables; (**b**) Distribution of imputation accuracy of highest missing continuous variables
